# A Novel Phosphorylation Site-Kinase Network-Based Method for the Accurate Prediction of Kinase-Substrate Relationships

**DOI:** 10.1155/2017/1826496

**Published:** 2017-10-12

**Authors:** Minghui Wang, Tao Wang, Binghua Wang, Yu Liu, Ao Li

**Affiliations:** ^1^School of Information Science and Technology, University of Science and Technology of China, 443 Huangshan Road, Hefei 230027, China; ^2^Research Centers for Biomedical Engineering, University of Science and Technology of China, 443 Huangshan Road, Hefei 230027, China

## Abstract

Protein phosphorylation is catalyzed by kinases which regulate many aspects that control death, movement, and cell growth. Identification of the phosphorylation site-specific kinase-substrate relationships (ssKSRs) is important for understanding cellular dynamics and provides a fundamental basis for further disease-related research and drug design. Although several computational methods have been developed, most of these methods mainly use local sequence of phosphorylation sites and protein-protein interactions (PPIs) to construct the prediction model. While phosphorylation presents very complicated processes and is usually involved in various biological mechanisms, the aforementioned information is not sufficient for accurate prediction. In this study, we propose a new and powerful computational approach named KSRPred for ssKSRs prediction, by introducing a novel phosphorylation site-kinase network (pSKN) profiles that can efficiently incorporate the relationships between various protein kinases and phosphorylation sites. The experimental results show that the pSKN profiles can efficiently improve the prediction performance in collaboration with local sequence and PPI information. Furthermore, we compare our method with the existing ssKSRs prediction tools and the results demonstrate that KSRPred can significantly improve the prediction performance compared with existing tools.

## 1. Introduction

As one of the most common posttranslational modifications (PTMs) [1, 2], phosphorylation plays an important role in the regulation of many cellular processes, such as signal transduction, translation, and transcription [3]. Phosphorylation is catalyzed by protein kinases and usually leads to a functional change, by changing cellular location, enzyme activity, or related to other proteins, of the target protein (substrate) [4, 5]. In human, nearly 75% of all proteins can be modified by protein kinases [6]. Abnormal activity of protein kinases often causes disease, especially cancer, in which protein kinases regulate many aspects that control death, movement, and cell growth [2, 7, 8]. On this point, identification of potential site-specific kinase-substrate relationships (ssKSRs) is important for understanding cellular dynamics and provides a fundamental basis for further disease-related researches and drug design.

To this end, several experimental methods, including low-throughput [9, 10] and high-throughput [11–13] biological technique, are developed to discover phosphorylation sites and corresponding kinases. However, low-throughput experimental identification employs one-by-one manner, which is not only time-consuming but also expensive. Although thousands of phosphorylation sites can be identified by high-throughput mass spectrometry (HTP-MS) techniques [13] in a single experiment [11, 12], it is still difficult to determine which of kinases is responsible for the phosphorylation of the observed site. Therefore, with large-scale phosphoproteomics studies, there is a huge gap between phosphorylation sites and protein kinases, which greatly hampers the study and elucidation of the mechanism of protein phosphorylation in signalling pathways.

So far, several computational methods [14–19] have been put forward to solve this problem during the past few decades, and most of them are mainly based on the sequence information. For example, Zou et al. [20] developed a web server, namely, PKIS, which adopts the composition of monomer spectrum (CMS) to encode the local sequence and then constructed the model with support vector machines (SVMs). Similarly, Damle and Mohanty et al. [15] develop an automated programmer called PhosNetConstruct for predicting target kinases for a substrate protein based on analysis of domain specific kinase-substrate relationships which are derived from the HMM profiles obtained from multiple sequence alignments of related proteins [15]. In addition, recently, some methods [17, 19] use protein-protein interactions (PPIs) to filter potential false positive to further improve performance. For example, Linding et al. [17] develop a web server, namely, NetworKIN, which is based on known sequence motif extracted from Scansite and NetPhosK, and the biological context of substrates is used as a filter to reduce false positives. Meanwhile, to discover the potential protein kinases of the unannotated phosphorylation sites, Song et al. develop a software package of iGPS [19], which is extended from GPS 2.0 [21] algorithm with the interaction filter.

Although these methods have achieved success, phosphorylation presents very complicated processes, it is usually involved in various biological mechanisms. In consequence, the aforementioned information adopted in the existing methods may not fully determine the corresponding protein kinase. It is well known that one protein kinase can catalyze multiple phosphorylation sites and one phosphorylation site can also be phosphorylated by multiple protein kinases [22–24]. For example, CDK2 can catalyze T8, T179, and S213 of protein SMAD3 (P84022), S567 of protein RB1 (P06400), and many other phosphorylation sites [25, 26]. Likewise, S315 of protein TP53 (P04637) can be catalyzed by AURKA, CDK1, CDK2, and so on [27, 28]. The relationships between various protein kinases and phosphorylation sites may bring valuable functional information of protein phosphorylation, which would be helpful in ssKSRs prediction in practice.

Inspired by this information, we propose a novel computational method in this study, namely, KSRPred, for ssKSRs prediction by introducing a phosphorylation site-kinase network (pSKN) profiles that can efficiently incorporate the relationships between various protein kinases and phosphorylation sites. This method is based on the framework of kernel ridge regression [29, 30], which can effectively integrate both pSKN profiles and other useful information including local sequences and PPIs. The experimental results show that the pSKN profiles can efficiently improve the prediction performance in collaboration with local sequence and PPI information. Furthermore, we compare KSRPred with the widely used ssKSRs prediction tools. The results also indicate that the proposed method has a better or comparable prediction performance compared with the existing ssKSRs prediction tools.

## 2. Materials and Methods

### 2.1. Data Collection and Preprocessing

In this study, we employ an experimentally verified human phosphorylation sites with corresponding kinases dataset, which include 6,839 verified sites and 389 kinases with 9,480 known ssKSRs extracted from Phospho.ELM [31] and the latest PhosphoSitePlus [32]. And, for this dataset, we follow Xu et al. [33] and Wang et al. [34] and use BlastClust with 70% threshold to remove substrate redundancy. Since iGPS [19], PKIS [20], and NetworKIN [17] use Phospho.ELM as training data, the phosphorylation sites existing in both training and testing data would overestimate the prediction performance. And for fair comparison with the existing tools, we extract an independent test dataset with 1,000 phosphorylation sites from the nonredundant dataset, which excludes the existing phosphorylation sites deposited in Phospho.ELM [31] and the rest as the training dataset. For a specific kinase, the verified sites modified by this kinase are taken as positive samples, and other verified sites are used as negative samples [35]. To achieve a reliable result [15, 36], here we construct models for kinases that at least 15 positive samples and finally 103 kinases are obtained. The detailed information of these kinases are summarized in Table S1 (see Supplementary Material available online at https://doi.org/10.1155/2017/1826496).

### 2.2. The Sequence Kernel Similarity

A local sequence with a length of 15 amino acids is extracted from the phosphorylation site, which contains 7 upstream and 7 downstream residues. We compute the sequence similarities of two phosphorylation sites using BLOSUM62 matrix, which is an amino acid substitution matrix that shows the similarities among 20 types of amino acids and usually used to calculate the sequence similarity [37]. The similarity between two phosphorylation sites *s*_*i*_ and *s*_*j*_ is calculated as follows:

(1) $$\begin{array}{c} S_{seq}\left(\;s_{i},s_{j}\;\right)=\overset{15}{\underset{k=1}{\sum }}BLOSUM62\left(\;s_{i}\left(\;k\;\right),s_{j}\left(\;k\;\right)\;\right), \end{array}$$

where BLOSUM62(*s*_*i*_(*k*), *s*_*j*_(*k*)) is the similarity score between the *k*th amino acid of *s*_*i*_ and the *k*th amino acid of *s*_*j*_ given by BLOSUM62 matrix. Applying this operation to all phosphorylation sites pairs, we construct a similarity matrix denoted as *S*_seq_. To ensure that the value of *S*_seq_ is distributed in the range of [0, 1], normalization is performed subsequently, and the formula is defined as *K*_seq_(*i*, *j*) = (*S*_seq_(*i*, *j*) − min⁡*S*_seq_)/(max⁡*S*_seq_ − min⁡*S*_seq_). The similarity matrix *K*_seq_ is considered as kernel similarity matrix of phosphorylation sites calculated from sequence level.

### 2.3. The PPI Kernel Similarity

The PPI information of substrates is extracted from STRING [38], which is a comprehensive, yet quality-controlled collection of protein-protein associations. Since these associations are derived from high-throughput experimental data, from the mining of database and literature and from predictions based on genomic context analysis [38], we follow Butland et al. [39] and Jafari et al. [40] and use a median (0.4) confidence cut-off value to filter the association. And 18,836 proteins that interacted with the 2,162 nonredundancy substrates are obtained. We compute the PPI similarities between two substrates using Jaccard Index [41]. The similarity between two substrates *p*_*i*_ and *p*_*j*_ is calculated as *S*_ppi_(*p*_*i*_, *p*_*j*_) = |*J*_*p*_*i*__∩*J*_*p*_*j*__|/|*J*_*p*_*i*__ ∪ *J*_*p*_*j*__|, where *J*_*p*_*i*__ and *J*_*p*_*j*__ represent the PPI information of corresponding substrate, respectively. Applying this operation to all substrate pairs, we construct a similarity matrix denoted as *S*_ppi_. However, some substrates have more than one phosphorylation sites; these sites have same substrates and share the same PPI information [42]. The similarity matrix *K*_ppi_ of phosphorylation sites can be obtained by directly extracting the similarity of substrates. The similarity matrix *K*_ppi_ is considered as kernel similarity matrix of phosphorylation sites calculated from substrate level.

### 2.4. Construction of pSKN Profiles and Kernel Similarity

**Figure 1:**
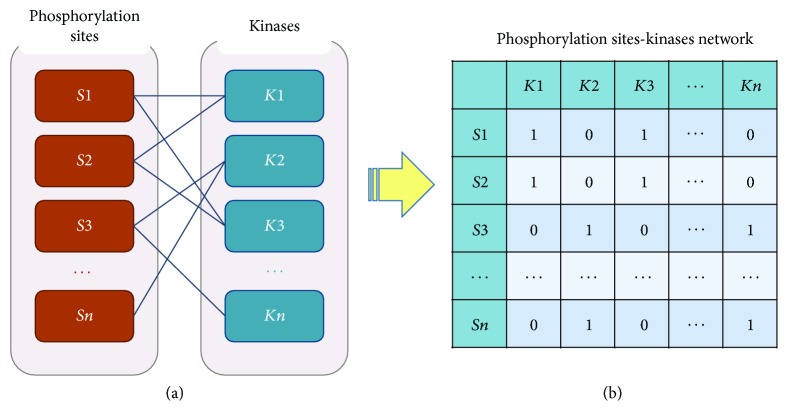
*Construction of phosphorylation site-kinase network and extracting the pSKN profiles*. The bipartite network represents the relationships between the phosphorylation sites and kinases; the orange and blue nodes represent phosphorylation sites and kinases, respectively. The matrix represents the pSKN profiles that are extracted from the bipartite network; each row is phosphorylation site *s*_*i*_ and each column is kinase *k*_*j*_; if *s*_*i*_ is catalyzed by *k*_*j*_, the value is 1, otherwise 0.

The relationships between various kinases and phosphorylation sites can be expressed as a bipartite network (Figure 1), from which we can extract a novel pSKN profiles. Formally, we denote the phosphorylation site set as *X*_*s*_ = {*s*_1_, *s*_2_,…, *s*_*n*_*s*__} and the kinase set as *X*_*k*_ = {*k*_1_, *k*_2_,…, *k*_*n*_*k*__}; the relationships between various kinases and phosphorylation sites can be described as a bipartite network *G*(*X*_*s*_, *X*_*k*_, *E*), where *E* = {*e*_*ij*_ : *s*_*i*_ ∈ *X*_*s*_, *k*_*j*_ ∈ *X*_*k*_}. A link is drawn between *s*_*i*_ and *k*_*j*_ when the phosphorylation site *s*_*i*_ has relationship with the kinase *k*_*j*_. This bipartite network can be presented by an *n*_*s*_ × *n*_*k*_ adjacent matrix *Y*, where *y*_*ij*_ = 1 if *s*_*i*_ and *k*_*j*_ are linked, while all other unknown phosphorylation site-kinase pairs are labeled as 0. Afterwards, to incorporate pSKN profiles for prediction, we construct a kernel similarity matrix from the pSKN profiles using Gaussian kernel function (i.e., RBF). The similarity between two phosphorylation sites *s*_*i*_ and *s*_*j*_ is calculated as follows:

(2) $$\begin{array}{c} K_{net}\left(\;s_{i},s_{j}\;\right)=\exp\left(\;-\gamma_{s}{\left\|\;y_{s_{i}}-y_{s_{j}}\;\right\|}^{2}\;\right), \end{array}$$

where *y*_*s*_*i*__ and *y*_*s*_*j*__ represent the *i*th and *j*th row of the adjacency matrix *Y*, respectively. The kernel bandwidth is controlled by the parameter *γ*_*s*_. It is normally defined as a new bandwidth parameter *γ*_*s*_′ normalized by the average number of relationships with phosphorylation site per kinase. The formula for the calculation of *γ*_*s*_ is

(3) $$\begin{array}{c} \gamma_{s}=\frac{\gamma_{s}^{\prime }}{\left(\left(1/n\right)\sum_{i=1}^{n}{\left\|y_{s_{i}}\right\|}^{2}\right)}. \end{array}$$

Applying this operation to all phosphorylation site pairs, we construct a similarity matrix denoted as *K*_net_. The similarity matrix *K*_net_ is considered as kernel similarity matrix of phosphorylation sites calculated from relationship level.

### 2.5. Kernel Ridge Classifier

To our knowledge, kernel ridge regression (KRR) is widely used in the field of bioinformatics [43–45], and existing studies [44] show that KRR and SVM have similar classification accuracy. In this study, we test these two algorithms on our dataset and find that KRR is comparable or slightly better than SVM. Therefore, we choose the KRR to construct the prediction model.

Formally, given a training dataset *T* = {(*x*_1_, *y*_1_),…, (*x*_*n*_, *y*_*n*_)}, where *x*_*i*_ ∈ *R*^*m*^ and *y*_*i*_ ∈ {0,1}, the basic idea of KRR relies on mapping the data into a higher dimensional space *ℋ* (also called feature space) according to a mapping Φ and then finding a linear regression function with the new training set *T* = {(Φ(*x*_1_), *y*_1_),…, (Φ(*x*_*n*_), *y*_*n*_), }, which represents a nonlinear regression in the original input space [46]. The linear ridge regression problem consists in minimizing the following cost:

(4) $$\begin{array}{c} L\left(\;\omega \;\right)=\overset{}{\underset{i}{\sum }}{\left\|\;y_{i}-\omega^{T}\phi \left(\;x_{i}\;\right)\;\right\|}^{2}+\lambda {\left\|\;\omega \;\right\|}^{2}, \end{array}$$

where *λ* is a regularization parameter used to control the trade-off between the bias and variance of the estimate. By calculating the derivative of this cost function [47], we can get the optimal solution *ω*^*∗*^ = *ϕ*(*ϕ*^*T*^*ϕ* + *λI*_*n*_)^−1^*Y*. Therefore, for a new unlabeled sample *x*, the predicted label *y* (i.e., *y* = *ω*^*T*^ · Φ(*x*)) can be calculated by the following formula:

(5) fx=YΦTΦ+λIn−1ΦTΦx=YK+λIn−1kx,

where *Y* is the vector of values *y*_*i*_ and *K*(*x*_*i*_, *x*_*j*_) = Φ(*x*_*i*_)^*T*^Φ(*x*_*j*_) is the kernel function.

In this study, we develop three similarity kernels, namely, sequence similarity kernel, PPI similarity kernel, and pSKN similarity kernel, from different data sources. In order to make full use of these kernels, we follow van Laarhoven et al. [48] and define a custom kernel function. The formula is defined as follows:

(6) $$\begin{array}{c} K\left(\;x_{i},x_{j}\;\right)=\overset{}{\underset{\varphi \in \left\{seq,ppi,net\right\}}{\sum }}\eta_{\varphi }K_{\varphi }\left(\;x_{i},x_{j}\;\right),\;\eta_{\varphi }\geq 0. \end{array}$$

And for the reported results of our evaluation, the unweighted average is adopted, that is, *η*_*φ*_ = 1/3, *φ* ∈ {seq, ppi, net}. Using (5) and (6), we can easily construct the corresponding model and make prediction for unlabeled phosphorylation sites. The model is implemented by the* scikit-learn* library (version 0.18) [49] in the* Python* environment.

### 2.6. Performance Evaluation

Following previous works [50, 51], we use 10-fold cross-validation to evaluate the prediction performance of classifier. The receiver operating characteristic (ROC) curve and the area under the curve (AUC) are used to calculate the average performance of 10-fold cross-validations. Meanwhile, in order to ensure the reliability, fairly, the commonly used measurement indexes are also adopted: specificity (Sp), sensitivity (Sn), Matthew's correlation coefficient (MCC), *F*-Measure (*F*1), and Precision (Pre). The formula is defined as follows:

(7) Sp=TNTN+FPSn=TPFN+TPPre=TPFP+TPF1=2×Pre×SnPre+SnMCC=TP×TN−FP×FNTN+FN×TN+FP×TP+FN×TP+FP.

TN and TP represent the number of positive and negative sites that are correctly predicted, commonly called true negative and true positive, respectively, while FN and FP represent the number of negative and positive sites that are wrong predicted, commonly called false negative and false positive, respectively. It is noteworthy that when the numbers of positive and negative set are significantly imbalanced, MCC can be used to obtain the balance quality.

## 3. Results

### 3.1. Evaluation of pSKN Profiles

**Figure 2:**
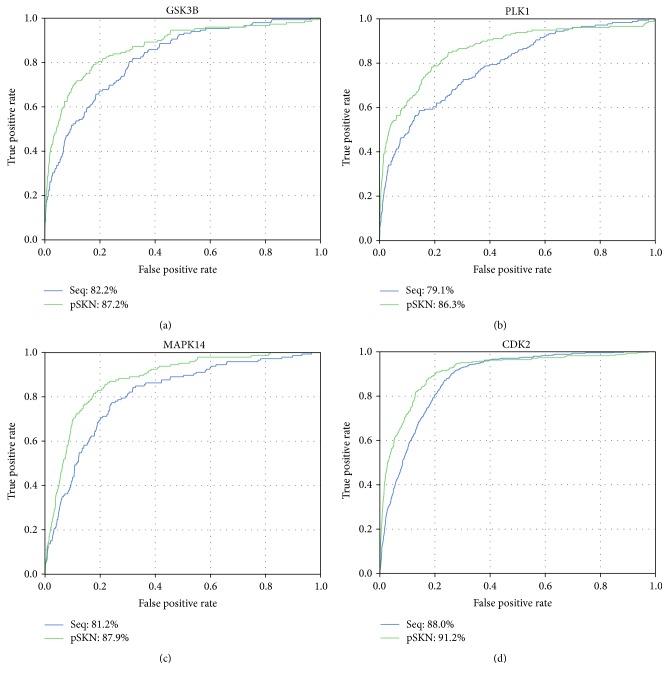
*Comparison of ROC curves using different information*. The blue lines represent our method constructed with local sequence only, and the green lines represent our method built with local sequence and pSKN profiles together.

In this study, we employ a novel pSKN profiles to predict ssKSRs. To confirm the effectiveness of pSKN profiles, we compare the proposed method with and without pSKN profiles on the basis of local sequence information. The prediction performances of these two methods are evaluated on the training dataset using 10-fold cross-validation. Here, we take kinase GSK3B, PLK1, P38A (MAPK14), and CDK2 as an example to illustrate the predictive performance, as shown in Figure 2. It is indicated that the proposed method with pSKN profiles shows a higher prediction accuracy in the ssKSRs prediction. For example, for GSK3B, the AUC value of the proposed method trained with local sequences is 82.2%. After applying pSKN profiles, the AUC value is improved to 87.2%, which is 5.0% higher than the proposed method trained with local sequences only. Likewise, for PLK1, compared to the proposed method with pSKN profiles and using local sequences only, the value of AUC is increased by 7.2%. Moreover, Figure S1 also displays the ROC curves of the three most pleiotropic protein kinases (i.e., PKCA, PKACA, and CK2A1), from which we can get a consistent conclusion. Taking PKCA as an example, the AUC value of our proposed method with pSKN profiles is 90.3%, which is 5.0% higher than the method with local sequences only.

Additionally, by following previous works [19, 20, 52], some measurements such as Sp, Sn, *F*1, Pre, and MCC are also adopted to ensure the reliability of performance evaluation. The measurements are evaluated at medium (Sp = 90.0%) and high (Sp = 95.0%) stringency levels, respectively.  Table 1 displays the Sn, *F*1, Pre, and MCC values of different kinases at medium stringency level. It is indicated that the proposed method with pSKN profiles achieves the best predictive performance in almost all cases. For example, for PKCA, the Sn, MCC, *F*1, and Pre values are 69.5%, 39.8%, 40.5%, and 28.6%, which are improved by 11.6%, 7.1%, 5.6%, and 3.6% compared with the method using local sequences only. Moreover, Table S2 displays the high stringency level of Sn, MCC, *F*1, and Pre values, from which we can draw a consistent conclusion. In all, these results show that pSKN profiles can significantly improve the prediction performance of different kinases.

Recently, several studies [17, 19] use the PPI information to filter false positive predictions, which can improve the precision of prediction results with the cost of reduced sensitivity [19]. Subsequently, we test the full method that integrates pSKN profile, local sequence, and PPI information to examine the ability of KSRPred in incorporating PPI information. The performance of AUC values and other measurements at high and medium stringency levels is listed in Table 1 and Table S2. As can be seen, for most of kinases, the proposed method can not only improve the precision of prediction results but also enhance the corresponding sensitivity, which indicates that the proposed method can make better use of PPI information in comparison with the existing methods [17, 19]. Taking P38A as an example, the AUC value of this full method is increased to 90.5%, which is 2.6% higher than the method with pSKN profiles. Besides, the Sn, MCC, *F*1, and Pre values at medium stringency level (Sp = 90.0%) are improved by 6.1%, 2.8%, 1.9%, and 1.1%, respectively. We also display the performance of other kinases in Table S3.

### 3.2. Comparison with the Existing ssKSRs Prediction Tools

**Figure 3:**
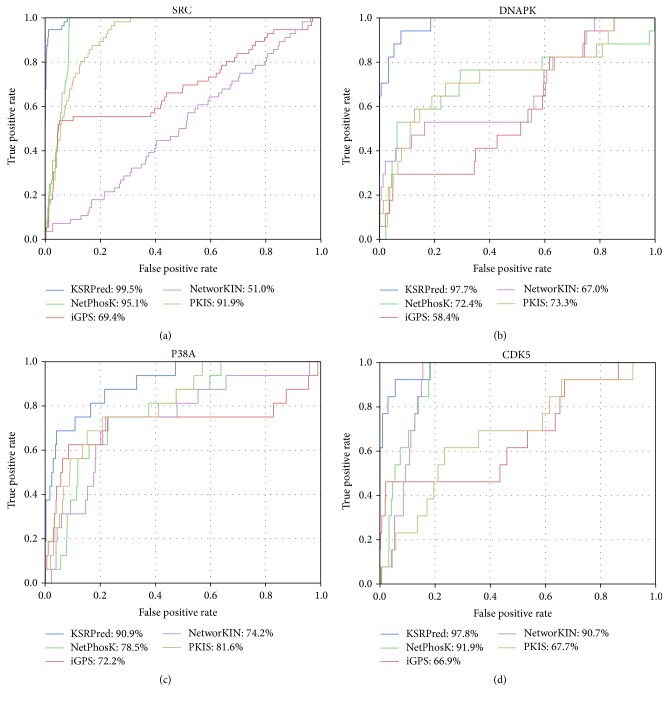
*Compare the ROC curves with different methods on the independent dataset*. The blue lines represent the ROC curve of KSRPred, and the green, red, purple, and yellow lines represent the ROC curves of NetPhosK, iGPS, NetworKIN, and PKIS, respectively.

In the previous section, we have verified the effectiveness of pSKN profiles. In this section, we use the independent test dataset to compare KSRPred with four widely used ssKSRs prediction tools, namely, NetPhosK [53], iGPS [19], NetworKIN [17], and PKIS [20], to evaluate the power of the proposed method. Here, we take four kinases that could be predicted by these tools as an example, and the corresponding ROC curves are displayed in Figure 3. It is indicated that the proposed method is generally superior to the existing tools. For example, for P38A, the AUC value of KSRPred is 90.9%, which is 12.4%, 18.7%, 16.7%, and 9.3% higher than those of NetPhosK, iGPS, NetworKIN, and PKIS, respectively. Likewise, for SRC, the AUC value of KSRPred is 4.40%, 30.10%, 48.50%, and 7.60% larger than those of NetPhosK, iGPS, NetworKIN, and PKIS, respectively.

**Figure 4:**
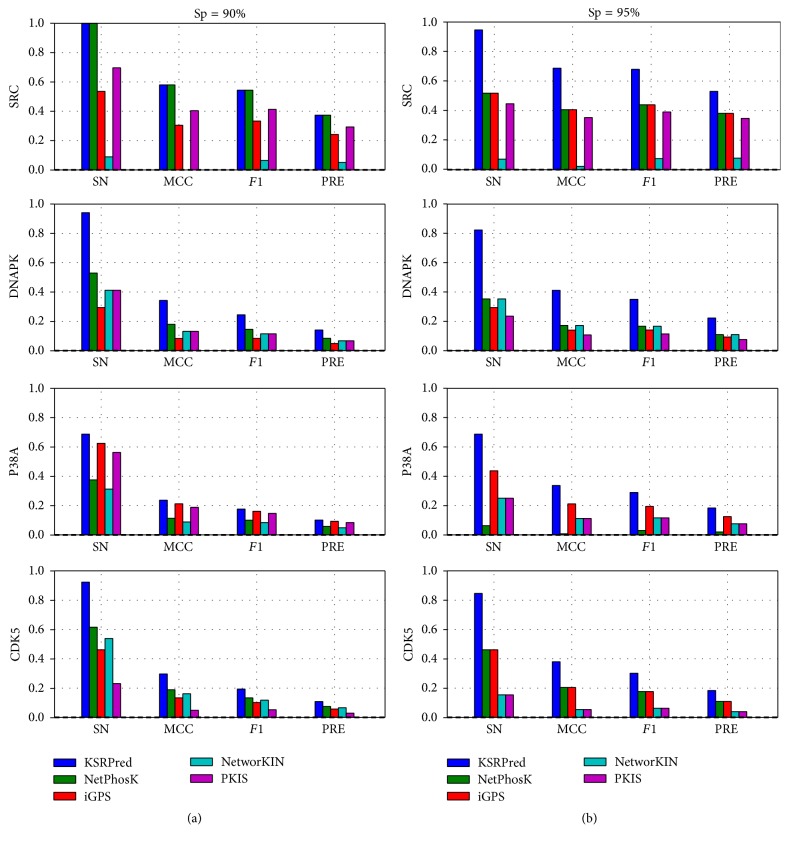
*Compare the Sn, MCC, F*1,* and Pre values of different methods on the independent dataset*. (a) represents the performance at specificity of 90.0%, and (b) represents the performance at specificity of 95.0%. The horizontal axis represents sensitivity, Matthew correlation coefficient, *F*1-measure, and precision, respectively.

In addition to the AUC values, the measurements (i.e., Sn, *F*1, Pre, and MCC) at medium and high stringency levels are also adopted to evaluate the performance. We draw the Sn-MCC-*F*1-Pre bar chart of the five methods based on the high and medium stringency levels, as shown in Figure 4 and the details are listed in Table S4. The experimental results show that KSRPred achieves the best performance in almost all circumstances in comparison with the existing tools. For example, for SRC, at the high stringency level, the Sn, MCC, *F*1, and Pre values of KSRPred are increased by 42.9%, 28.1%, 24.0%, and 14.8% compared with iGPS and have an improvement of 50.0%, 33.4%, 28.9%, and 18.3% compared with PKIS, respectively. Similarly, compared with NetPhosK and NetworKIN, the Sn, MCC, *F*1, and Pre values of KSRPred are also improved 42.9%, 28.1%, 24.0%, and 14.8% and 87.5%, 66.5%, 60.5%, and 45.2%, respectively. We further analyze the results of this kinase and find that at the high stringency level some phosphorylation sites can be correctly assigned by KSRPred, yet not by the existing tools. For example, Y53 of AKAP8 (O43823) is catalyzed by SRC and can be correctly assigned by our method but cannot be predicted by the existing tools. In summary, these results suggest that KSRPred achieves a better or comparable performance as compared with the existing ssKSRs prediction tools. In addition, in Figure S2, we also compare the performance of the proposed method without pSKN profile with NetPhosK and iGPS. The result shows that, compared with these two tools, KSRPred without pSKN profile can also get a better performance. Taken P38A as an example, the AUC achieved by KSRPred without pSKN profile is 7.8% and 14.1% higher than NetPhosK and iGPS, respectively.

### 3.3. Detailed Analysis of the Prediction Results

**Figure 5:**
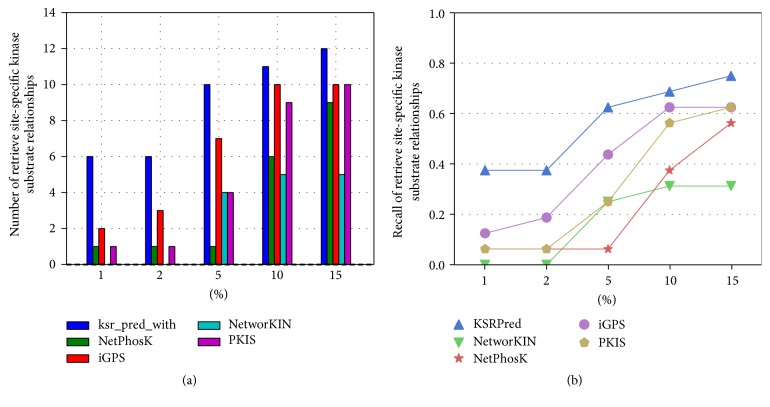
*Compare the ability of different methods in retrieve site-specific kinase-substrate relationships*. (a) represents the number of retrieved site-specific kinase-substrate relationships at the different percentiles, and (b) represents the fraction of retrieved site-specific kinase-substrate (recall).

After confirming the advantages of the proposed method, we conduct a detailed analysis on the prediction results. It is known that the predicted top-ranked results are more important in practice, which are utilized for proteomic-wide screening and systematic examination [42]. This requires the computational method with low false positive rate. Hence, we compare the numbers of correctly retrieved ssKSRs according to different percentiles. For each percentile *p*%, we count the number of true ssKSRs in the top-ranked *p*%*∗*1,000 predictions. Taking P38A as an example, results of five percentiles 1%, 2%, 5%, 10%, and 15% of the total phosphorylation sites number are compared, as shown in Figure 5. It is indicated that at all percentiles KSRPred can retrieve a more true positive prediction compared with NetPhosK, NetworKIN, iGPS, and PKIS.

In addition, due to the difficulty of experimental verification, computational method is also required to have the ability to detect unknown ssKSRs [42]. In view of this, we analyze the prediction result of top 20 potential phosphorylation sites. Taking CDK2 as an example, the detailed information of these phosphorylation sites is listed in Table 2. By mining of the literature, we find that some results have been confirmed as the phosphorylation sites catalyzed by this kinase. For example, Leng et al. [54] have reported that CDK2 can catalyze the S964 site of protein RBL1 (P28749). Likewise, from the UniProtKB database, we find that this kinase can catalyze the S975 site of protein RBL1 (P28749) (http://www.uniprot.org/uniprot/P28749#ptm_processing). These discoveries suggest that KSRPred has not only a lower false positive rate but also the ability to discover unknown ssKSRs, which could be helpful for the subsequent experimental verification.

## 4. Discussions and Conclusions

Phosphorylation plays a significant role in a wide range of cellular processes, which is catalyzed by protein kinases and many phosphorylation-related diseases are closely related to kinases. Prediction of ssKSRs is important for understanding phosphorylation process and provides a fundamental basis for further cell dynamics studies and drug design. However, traditional experimental methods are high-cost and time-consuming, and it is important to develop effective computational methods to predict ssKSRs. Although several computational methods for ssKSRs prediction have been proposed, these methods usually use the local sequence and PPI information, which are not sufficient for accurate prediction. In this study, we present the pSKN profiles that can efficiently incorporate the relationships between various kinases and phosphorylation sites. Using these pSKN profiles, the performance of our proposed method has been significantly improved. Meanwhile, we use PPIs extracted from STRING database as the substrate feature, and the experimental results show that our proposed method could make better use of this information compared with the existing method (e.g., iGPS and NetworKIN). Furthermore, through the analysis of potential phosphorylation sites, we find that some highly ranked results have been confirmed as phosphorylation sites catalyzed by kinases, suggesting its efficiency in discovering new potential ssKSRs for experimental validations and elucidating the molecular mechanism of protein phosphorylation.

Although the proposed method has shown the good ability for ssKSRs prediction, there is still much room for improvement. It is well known that the quantity of training data plays crucial roles in mastering the performance of machine learning methods [55, 56], and when more training data is available, the performance would be further improved. Additionally, kinases have corresponding family information and there are studies [33, 36] showing that this information is useful for ssKSRs prediction. In this study, we do not consider the influence of kinase family information, which can be integrated into the proposed method in further work. Moreover, the PPI dataset used in this study is from STRING database, and there are many other PPI databases that are publicly available, for example, MINT [57] and I2D [58], which can be included to further improve the performance of the proposed method. Furthermore, as kinase catalyzed phosphorylation site is a complex biological process affected by various mechanisms, incorporating more relevant functional information may also enhance the performance of ssKSRs prediction. Finally, the pSKN profiles are extracted from the relationships between kinases and phosphorylation sits, and the experimental results show that this information can effectively improve the prediction performance. However, available experimentally verified relationships between kinases and phosphorylation sits are still comparatively rare. Hence, it is expected that the performance of KSRPred will be further improved when more relationships can be obtained.

## Supplementary Material

Figure S1. Comparison of ROC curves using different information. Figure S2. Compare the ROC curves of three methods: KSRPred without pSKN profile, NetPhosK and iGPS. Table S1. The number of known phosphorylation sites of 103 kinases. Table S2. Comparison of prediction performance using different information at the high strategic level (Sp = 95.0%). Table S3. The predictive performance of all kinases. Table S4. Comparison of prediction performance of different methods at the high and medium strategy levels.

## Figures and Tables

**Table 1:** Compare the predictive performance of our methods using different information at medium stringency level (Sp = 90.0%).

Kinases	Methods	AUC	Sn	MCC	*F*1	Pre
CDK2	Seq	88.0%	55.9%	35.8%	40.5%	31.8%
	pSKN	91.2%	72.2%	46.7%	49.4%	37.5%
	Full	93.4%	83.1%	53.6%	54.8%	40.9%

CK2A1	Seq	93.0%	83.4%	50.1%	49.8%	35.5%
	pSKN	94.3%	86.1%	51.8%	51.0%	36.2%
	Full	94.4%	88.4%	53.1%	52.0%	36.8%

FYN	Seq	93.3%	74.1%	24.6%	17.4%	9.9%
	pSKN	94.6%	83.5%	28.1%	19.4%	11.0%
	Full	95.5%	84.7%	28.5%	19.7%	11.1%

GSK3B	Seq	82.2%	51.7%	21.0%	19.4%	11.9%
	pSKN	87.2%	68.5%	28.9%	24.9%	15.2%
	Full	89.3%	73.8%	31.4%	26.6%	16.2%

P38A	Seq	81.2%	43.2%	16.7%	16.2%	10.0%
	pSKN	87.9%	69.2%	29.0%	24.8%	15.1%
	Full	90.5%	75.3%	31.8%	26.7%	16.2%

PKACA	Seq	90.1%	70.5%	41.5%	42.5%	30.5%
	pSKN	91.9%	77.2%	45.5%	45.7%	32.4%
	Full	93.0%	81.0%	47.8%	47.4%	33.5%

PKCA	Seq	85.3%	57.9%	32.7%	34.9%	25.0%
	pSKN	90.3%	69.5%	39.8%	40.5%	28.6%
	Full	91.5%	80.2%	46.2%	45.3%	31.6%

PLK1	Seq	79.1%	48.0%	20.8%	20.7%	13.2%
	pSKN	86.3%	62.6%	28.3%	26.1%	16.5%
	Full	89.7%	80.4%	37.2%	32.4%	20.3%

SRC	Seq	94.5%	88.3%	51.1%	49.3%	34.2%
	pSKN	96.1%	86.4%	50.1%	48.5%	33.7%
	Full	97.2%	92.9%	53.8%	51.2%	35.3%

**Table 2:** Information of top 20 potential phosphorylation sites for CDK2 kinase.

Ranking	UniProtKB	Protein name	Site	Score
1	Q08999	RBL2	S1035	0.4707
2	P28749	RBL1	S964	0.4672
3	P28749	RBL1	T369	0.4481
4	Q08999	RBL2	S672	0.4403
5	P28749	RBL1	S975	0.4389
6	Q08999	RBL2	T401	0.4313
7	Q9UQ35	SRRM2	T1413	0.3952
8	P49736	MCM2	S31	0.3736
9	Q9Y5N6	ORC6	T195	0.3717
10	P24928	POLR2A	S1878	0.3663
11	Q15910	EZH2	T487	0.3653
12	Q9UQ35	SRRM2	T866	0.3553
13	O15446	CD3EAP	S285	0.3505
14	P24928	POLR2A	S1920	0.3495
15	P24928	POLR2A	S1934	0.3492
16	Q02539	HIST1H1A	S183	0.3488
17	P10276	RARA	S77	0.3425
18	Q5TKA1	LIN9	T96	0.3412
19	Q9P1Z0	ZBTB4	T983	0.3347
20	P49736	MCM2	T59	0.3338
