# Supplementary material for: A Novel Phosphorylation Site-Kinase Network-Based Method for the Accurate Prediction of Kinase-Substrate Relationships
Source: Biomed Res Int. 2017 Oct 12;2017:1826496. doi: 10.1155/2017/1826496 (PMC5660750; doi:10.1155/2017/1826496)
Supplement: Supplementary file 1 — Figure S1. Comparison of ROC curves using different information. Figure S2. Compare the ROC curves of three methods: KSRPred without pSKN profile, NetPhosK and iGPS. Table S1. The number of known phosphorylation sites of 103 kinases. Table S2. Comparison of prediction performance using different information at the high strategic level (Sp = 95.0%). Table S3. The predictive performance of all kinases. Table S4. Comparison of prediction performance of different methods at the high and medium strategy levels. [file 1826496.f1.pdf]

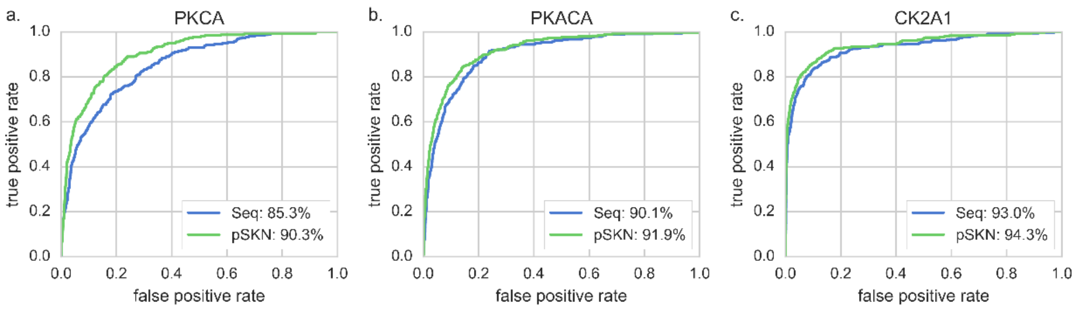

Figure S1. Comparison of ROC curves using different information. The blue lines represent our method constructed with local sequence only, and the green lines represent our method built with local sequence and pSKN profiles together.

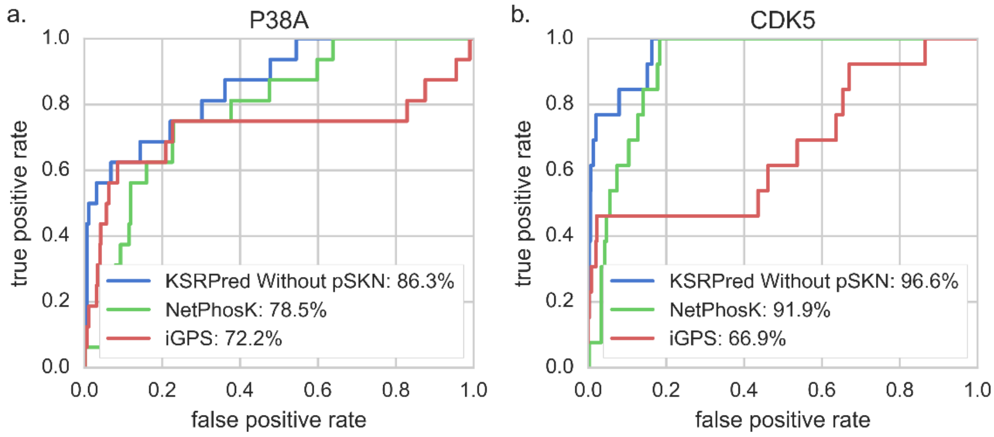

Figure S2. Compare the ROC curves of three methods: KSRPred without pSKN profile, NetPhosK and iGPS. The blue lines represent the ROC curve of KSRPred without pSKN profile, and the red and green lines represent the ROC curves of iGPS and NetPhosK, respectively.

Table S1. The number of known phosphorylation sites of 103 kinases.

Kinases	Number	Kinases	Number	Kinases	Number	Kinases	Number
ABL	112	CK1E	27	JAK2	29	PKCA	318
AKT1	182	CK2A1	361	JAK3	16	PKCB	33
AKT2	19	CK2A2	17	JNK1	86	PKCB ISO2	30
AMPKA1	68	DAPK3	15	JNK2	31	PKCD	80
AMPKA2	19	DNAPK	88	LCK	78	PKCE	47
ASK1	16	DYRK1A	18	LKB1	31	PKCG	20
ATM	193	EGFR	59	LRRK2	46	PKCI	16
ATR	67	ERK1	214	LYN	76	PKCT	26
AURA	75	ERK2	269	MAPKAPK2	53	PKCZ	46
AURB	114	ERK5	21	MELK	22	PKG1	33
BTK	19	ETK	16	MET	29	PKR	17
BUB1	23	FAK	19	MSK1	15	PLK1	179
CAMK2A	69	FGFR1	31	MST1	21	PLK3	28
CDC7	18	FYN	85	MST2	19	PRKD1	44
CDK1	401	GRK2	50	MTOR	67	RET	24
CDK2	449	GSK3A	20	NEK2	21	ROCK1	47
CDK4	44	GSK3B	149	P38A	146	RSK2	41
CDK5	73	HCK	20	P38B	20	SGK1	23
CDK6	24	HER2	15	P70S6K	33	SRC	324
CDK7	39	HIPK2	33	P90RSK	48	SRPK1	16
CDK9	34	IGF1R	19	PAK1	54	SYK	49
CHAK1	41	IKKA	26	PAK2	17	TBK1	26
CHK1	149	IKKB	41	PDGFRB	22	TTK	41
CHK2	48	IKKE	21	PDK1	28	ULK1	18
CK1A	85	INSR	39	PIM1	29	ZAP70	20
CK1D	54	IRAK4	18	PKACA	342		

Table S2. Comparison of prediction performance using different information at the high strategic level (*Sp* = 95.0%).

Kinases	Methods	*Sn*	*MCC*	*F1*	*Pre*
CDK2	*Seq*	38.3%	33.5%	38.6%	38.9%
	pSKN	57.5%	48.7%	52.8%	48.9%
	Full	71.9%	59.0%	62.0%	54.5%
CK2A1	*Seq*	74.8%	57.8%	59.7%	49.6%
	pSKN	79.8%	61.1%	62.4%	51.2%
	Full	83.9%	63.7%	64.6%	52.5%
FYN	*Seq*	44.7%	20.7%	18.5%	11.7%
	pSKN	58.8%	27.6%	23.6%	14.8%
	Full	69.4%	32.6%	27.3%	17.0%
GSK3B	*Seq*	34.9%	20.2%	21.4%	15.4%
	pSKN	52.3%	30.9%	30.5%	21.5%
	Full	58.4%	34.5%	33.4%	23.4%
P38A	*Seq*	27.4%	15.2%	17.0%	12.3%
	pSKN	39.7%	23.1%	23.7%	16.9%
	Full	47.9%	28.1%	27.9%	19.7%
PKACA	*Seq*	53.2%	42.2%	45.6%	39.8%
	pSKN	62.0%	48.4%	51.1%	43.5%
	Full	70.2%	54.0%	56.0%	46.6%
PKCA	*Seq*	45.6%	35.6%	39.2%	34.4%
	pSKN	58.8%	45.2%	47.9%	40.4%
	Full	63.8%	48.7%	50.9%	42.4%
PLK1	*Seq*	37.4%	23.6%	25.3%	19.1%
	pSKN	54.2%	34.4%	34.7%	25.5%
	Full	66.5%	41.9%	41.0%	29.6%
SRC	*Seq*	46.9%	36.8%	40.4%	35.5%
	pSKN	72.2%	54.5%	56.1%	45.9%
	Full	78.7%	58.7%	59.6%	48.0%

Table S3. The predictive performance of all kinases

Kinases	*Full*	Kinases	*Full*	Kinases	*Full*	Kinases	*Full*
ABL	95.7%	CK1E	96.7%	JAK2	94.4%	PKCA	90.9%
AKT1	95.1%	CK2A1	94.2%	JAK3	99.4%	PKCB	85.2%
AKT2	88.0%	CK2A2	98.0%	JNK1	93.9%	PKCB ISO2	91.7%
AMPKA1	91.4%	DAPK3	89.9%	JNK2	90.3%	PKCD	84.2%
AMPKA2	87.9%	DNAPK	93.7%	LCK	96.3%	PKCE	84.7%
ASK1	98.1%	DYRK1A	90.5%	LKB1	93.6%	PKCG	85.5%
ATM	95.6%	EGFR	95.5%	LRRK2	96.9%	PKCI	86.2%
ATR	96.1%	ERK1	95.7%	LYN	95.4%	PKCT	79.6%
AURA	88.7%	ERK2	95.1%	MAPKAPK2	85.7%	PKCZ	87.4%
AURB	94.1%	ERK5	90.6%	MELK	96.4%	PKG1	94.9%
BTK	95.8%	ETK	98.9%	MET	99.5%	PKR	92.9%
BUB1	99.6%	FAK	91.1%	MSK1	87.3%	PLK1	89.7%
CAMK2A	85.3%	FGFR1	96.1%	MST1	96.7%	PLK3	85.4%
CDC7	99.5%	FYN	95.1%	MST2	99.1%	PRKD1	90.2%
CDK1	93.9%	GRK2	89.5%	MTOR	92.0%	RET	92.3%
CDK2	93.1%	GSK3A	93.9%	NEK2	95.2%	ROCK1	95.4%
CDK4	98.5%	GSK3B	89.0%	P38A	90.1%	RSK2	90.9%
CDK5	89.4%	HCK	97.8%	P38B	97.6%	SGK1	90.0%
CDK6	97.0%	HER2	90.6%	P70S6K	92.3%	SRC	97.1%
CDK7	91.0%	HIPK2	90.8%	P90RSK	90.3%	SRPK1	99.7%
CDK9	91.1%	IGF1R	98.8%	PAK1	79.5%	SYK	96.3%
CHAK1	99.5%	IKKA	93.3%	PAK2	92.1%	TBK1	92.0%
CHK1	92.2%	IKKB	92.9%	PDGFRB	96.1%	TTK	90.5%
CHK2	83.5%	IKKE	94.1%	PDK1	97.1%	ULK1	98.3%
CK1A	91.5%	INSR	97.0%	PIM1	91.5%	ZAP70	98.9%
CK1D	92.7%	IRAK4	99.9%	PKACA	92.4%		

Table S4. Comparison of prediction performance of different methods at the high and medium strategy levels.

**Kinases**	***Measures***	***Specificity (Sp)=95.0%***					***Specificity (Sp)=90.0%***				
		iGPS	***PKIS***	***NetworKIN***	***NetPhosK***	***KSRPred***	***iGPS***	***PKIS***	***NetworKIN***	***NetPhosK***	***KSRPred***
SRC	*Sensitivity (Sn)*	51.8%	44.6%	7.1%	51.8%	94.6%	53.6%	69.6%	8.9%	100.0%	100.0%
	F1-Measure (F1)	43.9%	39.1%	7.5%	43.9%	67.9%	33.3%	41.3%	6.5%	54.4%	54.4%
	Precision (Pre)	38.2%	34.7%	7.8%	38.2%	53.0%	24.2%	29.3%	5.1%	37.3%	37.3%
	Matthew's Correlation Coefficient (MCC)	40.6%	35.3%	2.3%	40.6%	68.7%	30.4%	40.4%	-0.8%	58.0%	58.0%
DNAPK	*Sensitivity (Sn)*	29.4%	23.5%	35.3%	35.3%	82.4%	29.4%	41.2%	41.2%	52.9%	94.1%
	F1-Measure (F1)	14.1%	11.4%	16.7%	16.7%	35.0%	8.3%	11.5%	11.5%	14.5%	24.4%
	Precision (Pre)	9.3%	7.5%	10.9%	10.9%	22.2%	4.9%	6.7%	6.7%	8.4%	14.0%
	Matthew's Correlation Coefficient (MCC)	14.0%	10.7%	17.2%	17.2%	41.2%	8.3%	13.2%	13.2%	18.0%	34.2%
P38A	*Sensitivity (Sn)*	43.8%	25.0%	25.0%	6.3%	68.8%	62.5%	56.3%	31.3%	37.5%	68.8%
	F1-Measure (F1)	19.4%	11.6%	11.6%	3.0%	28.9%	16.1%	14.6%	8.4%	10.0%	17.6%
	Precision (Pre)	12.5%	7.5%	7.5%	2.0%	18.3%	9.3%	8.4%	4.9%	5.8%	10.1%
	Matthew's Correlation Coefficient (MCC)	21.2%	11.2%	11.2%	0.7%	33.7%	21.2%	18.8%	8.8%	11.3%	23.7%
CDK5	*Sensitivity (Sn)*	46.2%	15.4%	15.4%	46.2%	84.6%	46.2%	23.1%	53.8%	61.5%	92.3%
	F1-Measure (F1)	17.6%	6.3%	6.3%	17.6%	30.1%	10.2%	5.2%	11.8%	13.3%	19.4%
	Precision (Pre)	10.9%	3.9%	3.9%	10.9%	18.3%	5.7%	2.9%	6.6%	7.5%	10.8%
	Matthew's Correlation Coefficient (MCC)	20.5%	5.4%	5.4%	20.5%	38.0%	13.3%	4.9%	16.1%	18.9%	29.7%
